# Pyridostigmine and low-dose naltrexone for ME/CFS: study protocol for the Life Improvement Trial (LIFT), a randomized, double-blind, placebo-controlled clinical trial

**DOI:** 10.1186/s13063-026-09943-6

**Published:** 2026-08-12

**Authors:** Danielle Meadows, Johanna Squires, Joshua Dibble, Shreya Palwayi, Donna Felsenstein, Linda Tannenbaum, Peng Li, Wenzhong Xiao, Jonas Bergquist, David Systrom

**Affiliations:** 1 Open Medicine Foundation, 29302 Laro Dr, Agoura Hills, CA 91301 USA; 2 Division of Pulmonary and Critical Care Medicine, Department of Medicine, Brigham and Women’s Hospital, Harvard Medical School, Boston, MA 02115 USA; 3 Innovation Center for Computational Health, Massachusetts General Hospital, Harvard Medical School, Boston, MA 02114 USA; 4 Department of Infectious Diseases, Massachusetts General Hospital, Harvard Medical School, Boston, MA 02114 USA; 5 Analytical Chemistry and Neurochemistry, Department of Chemistry—BMC, Uppsala University, 751 24, Uppsala, Sweden; 6 The ME/CFS Collaborative Research Centre at Uppsala University, 751 24, Uppsala, Sweden

**Keywords:** ME/CFS, LDN, Pyridostigmine, Mestinon, FUNCAP-55, Garmin, PROMIS-29, DSQ-PEM, SHAPE

## Abstract

**Background:**

Myalgic encephalomyelitis/chronic fatigue syndrome (ME/CFS) is a complex, chronic disease with no FDA-approved treatments. This report describes a protocol for the Life Improvement Trial (LIFT), a randomized, double-blind, placebo-controlled clinical trial investigating the impact of low-dose naltrexone (LDN) and pyridostigmine (Mestinon) on physiological response, symptoms, and functionality of ME/CFS patients.

**Methods:**

Participants (target *n* = 160) are recruited through clinics at Massachusetts General Hospital and Brigham and Women’s Hospital, and through Open Medicine Foundation’s StudyME registry. They are then randomized into one of four arms: LDN/pyridostigmine, LDN/placebo, placebo/pyridostigmine, placebo/placebo. Treatment is administered for 13 weeks after an initial screening period of up to 4 weeks. Primary outcomes are FUNCAP-55 score, peak oxygen utilization, heart rate recovery, and oxygen uptake efficiency slope. Secondary outcomes are scores from DSQ-PEM and PROMIS-29 surveys, DANA Brain Vital score, step count, heart rate, and heart rate variability.

**Discussion:**

The results of this trial will provide novel insights into the efficacy of and predictors of response to LDN and pyridostigmine in ME/CFS. This may inform future treatment strategies for ME/CFS. The trial will also validate what primary and secondary outcomes to use in similar clinical trials.

**Trial registration:**

This trial was registered on clinicaltrials.gov (NCT06366724) on April 16, 2024.

**Supplementary Information:**

The online version contains supplementary material available at https://doi.org/10.1186/s13063-026-09943-6.

## Introduction

### Background and rationale

Myalgic encephalomyelitis/chronic fatigue syndrome (ME/CFS) is a chronic, debilitating, complex disease that impacts multiple systems in the body, including the immune, metabolic, cardiovascular, and nervous systems [1]. Its diagnosis relies on identifying hallmark symptoms such as profound fatigue, unrefreshing sleep, orthostatic intolerance, neurocognitive disturbances and brain fog, pain, and post-exertional malaise (PEM)—the worsening of symptoms following even mild physical or cognitive exertion [2]. Given pre-COVID-19 prevalence estimates [3] and the incidence of ME/CFS resulting from Long COVID [4, 5], it is estimated that ME/CFS exhibits a lifetime prevalence of over 275 million people worldwide. Despite the global impact of this condition, there are no FDA-approved treatments for ME/CFS, so clinical trials to identify efficacious interventions are critically needed [1].

Pyridostigmine, or Mestinon, and low-dose naltrexone (LDN) are two drugs that have shown promise in the treatment of ME/CFS. Pyridostigmine is an acetylcholinesterase inhibitor, which can increase the level and duration of acetylcholine in the neural synapse [6, 7]. It is currently FDA-approved for the treatment of myasthenia gravis [8]. Naltrexone is an approved treatment for alcohol and/or opioid use disorder, which has anti-inflammatory effects at low doses (0.5–4.5 mg), making LDN relevant for the treatment of ME/CFS [9]. Pyridostigmine and LDN address different underpinnings of symptoms in ME/CFS: neurovascular dysregulation and inflammation, respectively. The combination of the two drugs has never been studied.

People with ME/CFS often exhibit vascular dysregulation, with low biventricular filling pressures (preload failure) resulting in decreased aerobic capacity [10]. By increasing levels of acetylcholine, it is thought that pyridostigmine increases cholinergic stimulation of norepinephrine release at the postganglionic synapse, which, in turn, improves constriction of capacitance vessels and return of blood to the heart. This may then lead to improved right atrial pressure in ME/CFS patients [11].

Previous work has demonstrated that pyridostigmine can improve aerobic capacity in ME/CFS [12]. As measured by a submaximum exercise heart and pulmonary evaluation (SHAPE) test, pyridostigmine treatment increases oxygen uptake efficiency and pulmonary vascular capacitance [12]. In postural orthostatic tachycardia syndrome (POTS), a common comorbidity of ME/CFS, pyridostigmine has been shown to reduce standing heart rate, improve diastolic blood pressure, and significantly decrease symptom burden after acute use [13, 14]. Acetylcholinesterase inhibitors are also used to improve patients’ orthostatic intolerance, which is an important symptom of ME/CFS [15]. There is limited evidence on the long-term efficacy of pyridostigmine, however, so structured clinical trials are required.

In addition to vascular dysregulation and orthostatic intolerance, chronic inflammation is a common occurrence in ME/CFS [9]. LDN is thought to address this by modulating glial cells and peripheral blood mononuclear cells as a toll-like receptor (TLR) antagonist [9, 16–19]. Through TLR-4 regulation and modulation of the response to intracellular TLR activity, LDN reduces pro-inflammatory cytokines like interleukin-1, interleukin-6, tumor necrosis factor-alpha, interferon beta, and nitric oxide [9, 16]. This anti-inflammatory effect thus reduces fatigue, inflammation, pain, and cognitive issues [9, 17, 19].

In the clinic, LDN treatment has produced positive responses in people with ME/CFS [20] and similar diseases, including fibromyalgia [21, 22], multiple sclerosis, irritable bowel syndrome, and Crohn’s disease [9, 23, 24]. A retrospective analysis of 218 ME/CFS patients in Finland found that 73.9% of people experienced less pain and improved physical and cognitive performance after LDN treatment [23]. In Long COVID—a disease with overlapping symptomology and potentially pathophysiology as ME/CFS [25]—a 2-month treatment period with LDN was shown to reduce symptom burden, including pain levels, sleep disturbance, and limitation in daily living activities [26]. The majority of prospective evidence on the efficacy of LDN in ME/CFS and related conditions is subjective with limited sample sizes, however, so large trials with objective outcomes are necessary.

### Objectives

This clinical trial, designated as the Life Improvement Trial (LIFT), aims to evaluate the effect of pyridostigmine and LDN on the functional capacity, physiological response, and symptoms of ME/CFS via a randomized, double-blind, placebo-controlled study design. This is the first known investigation of potential additive effects of using the two drugs in combination. As there are no approved treatments for ME/CFS or standard of care, the trial aims to show superiority over placebo, the chosen comparator. The trial will also evaluate any potential differences between responders and non-responders. Ultimately, the LIFT will provide rigorous scientific evidence of the clinical utility of pyridostigmine and LDN, which may lead to more personalized treatment plans for people with ME/CFS in the future.

## Methods

This report follows SPIRIT reporting guidelines [27]. A completed SPIRIT checklist is provided as supplementary material.

### Trial design overview

The LIFT is a randomized, double-blind, placebo-controlled clinical trial. The study was approved by the Institutional Review Board of Massachusetts General Brigham (2024P000087). In-person recruitment is through clinics at Massachusetts General Hospital and Brigham and Women’s Hospital (BWH). Further recruitment is through Open Medicine Foundation’s StudyME registry.

**Fig. 1:**
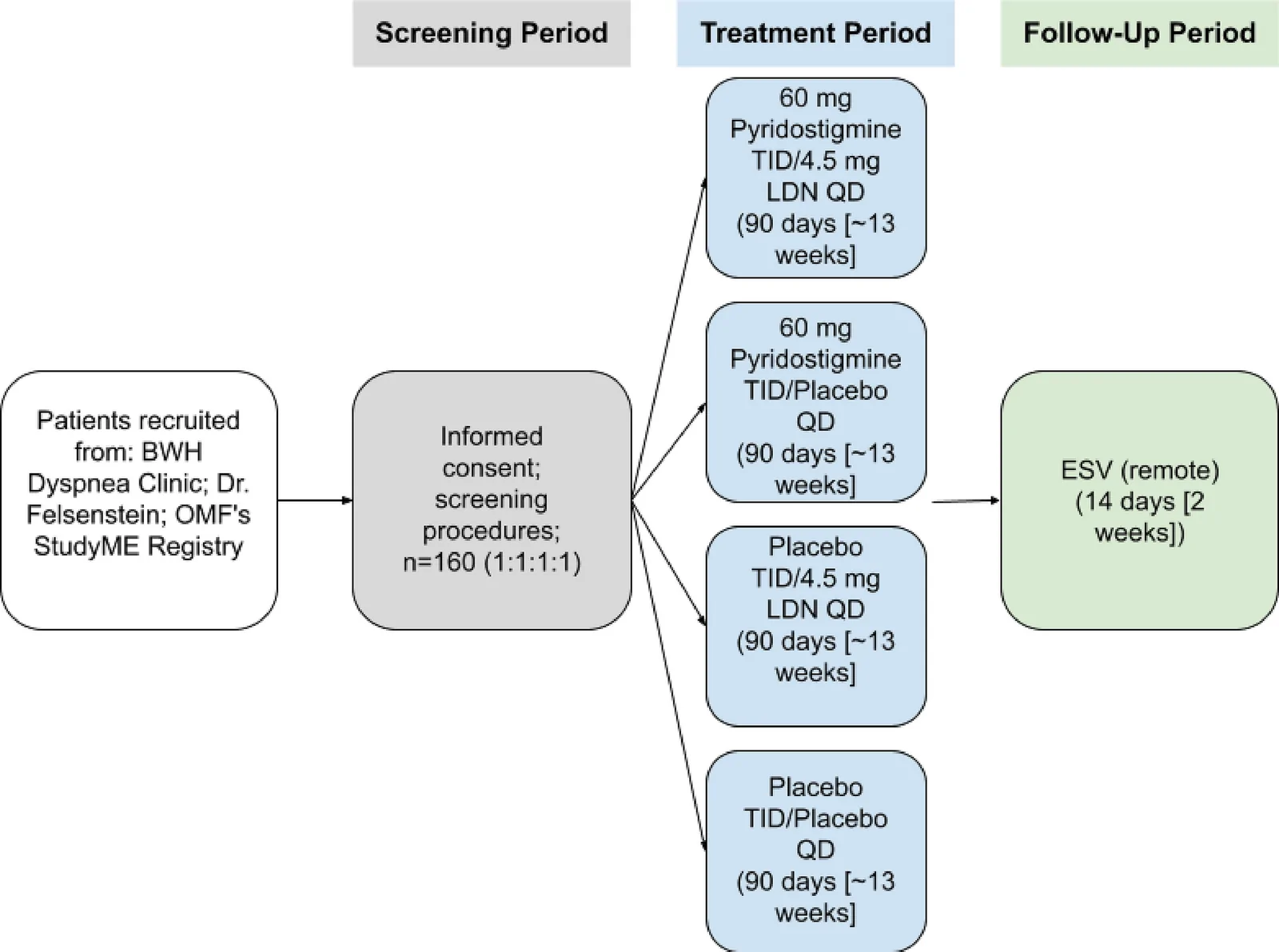
Study schema. Participants are recruited from the BWH dyspnea clinic, Open Medicine Foundation’s StudyME registry, and the co-investigator’s clinic. They are then screened, and if deemed eligible, they provide informed consent before being randomized 1:1:1:1 into four treatment groups: pyridostigmine and LDN, pyridostigmine and placebo, placebo and LDN, placebo and placebo. The treatment period lasts for 90 days, after which they will complete an end-of-study visit (ESV)

Participants are randomized (1:1:1:1) to placebo/placebo, placebo/LDN, pyridostigmine/placebo, and pyridostigmine/LDN arms. After 4 weeks of titration, 60 mg of pyridostigmine will be taken three times a day and 4.5 mg of low-dose naltrexone will be taken once a day. Randomization is conducted by the BWH Investigational Drug Services (IDS), and arm assignment is not shared with trial personnel or participants. Randomization is stratified by sex (male/female), and the randomization code will not be released by the pharmacy until the completion of the trial to ensure allocation concealment. As a result, all assessments will be completed by staff blind to treatment allocation. The full study schema is shown in Fig. 1.

**Fig. 2:**
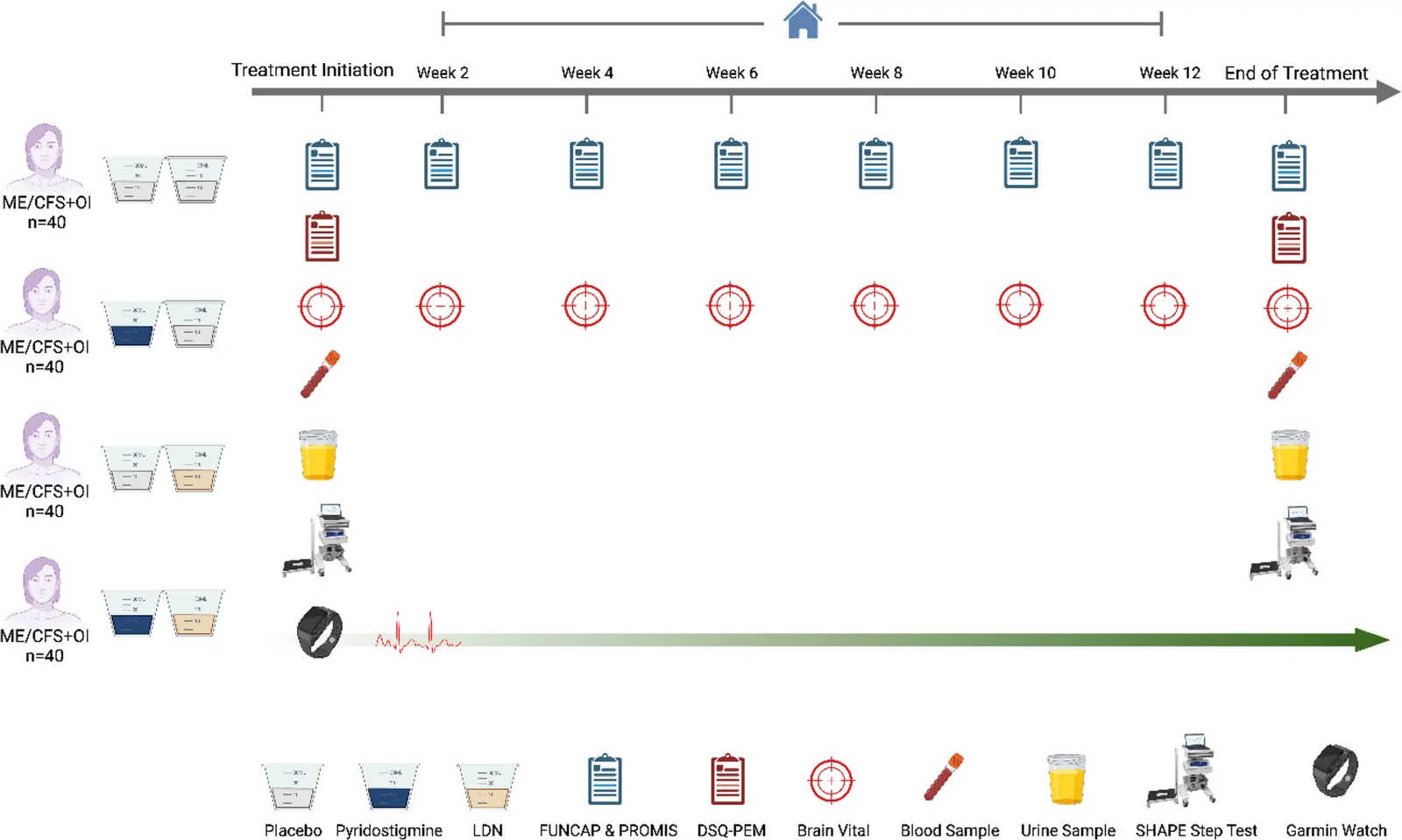
Overview of data collection during treatment period. Participants are randomized into four treatment arms: placebo/placebo, pyridostigmine/placebo, placebo/LDN, pyridostigmine/LDN. During treatment initiation, patient-reported outcomes, blood and urine samples, and Brain Vital data are collected. A SHAPE test is also conducted and the participant receives a Garmin watch. These are all also done at the end of treatment. Brain Vital, FUNCAP, and PROMIS will also be collected throughout the treatment period (created in BioRender.com)

All participants receive a Garmin Vivosmart® 5 watch at no cost. Data from Garmin watches are collected through Labfront [28]. Participant-reported outcomes (i.e., surveys) are completed through the REDCap web application. All participants also use the DANA Modular mobile application [29] to complete Brain Vital cognitive assessments. See Fig. 2 for a full overview of data collection throughout the treatment period.

**Fig. 3:**
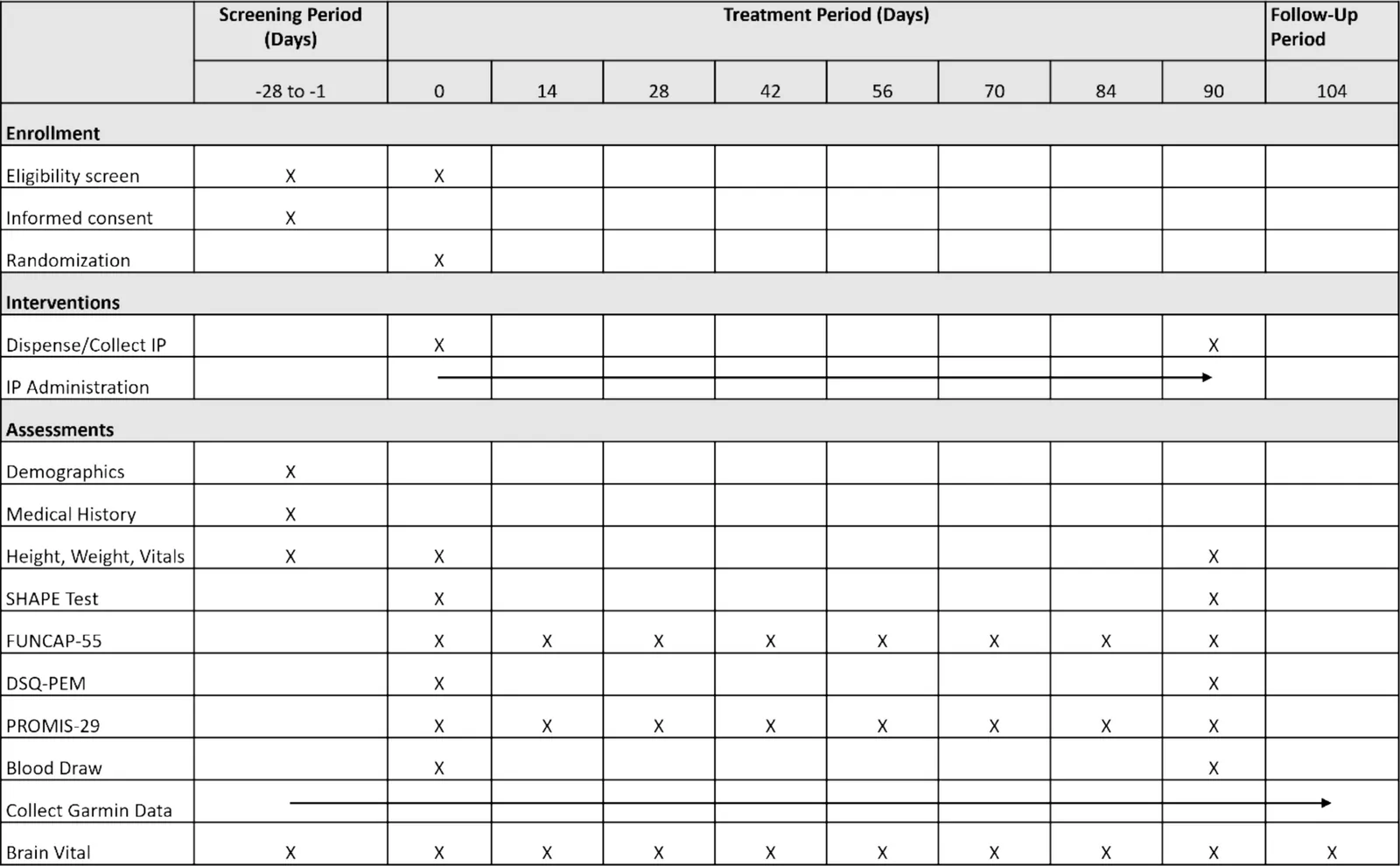
Schedule of enrollment, interventions, and assessments. Enrollment activities, intervention-related study tasks, and assessments are completed at each indicated time throughout the trial period. IP Administration and Collect Garmin Data are done daily during the time period spanned by each arrow, rather than at discrete weeks

Participants attend three in-person visits at the Clinical Trials Hub at Brigham and Women’s Hospital: for screening, to initiate the treatment period, and at the conclusion of the study 13 weeks later. A SHAPE test [30–32] and biosample collection (blood draw and urine) are performed during the two latter in-person visits. Blood samples are frozen and stored at −80 °C for future analyses, including proteomics and metabolomics, that might be used to determine if there are measurable differences between responders and non-responders. Coordinators also conduct periodic virtual visits with participants throughout the treatment period. For the full study schedule, see Fig. 3.

### Patient representative involvement

Four patient representatives were asked to provide feedback on the LIFT protocol. Their review, in addition to input from the larger patient community, directly resulted in protocol updates, including expanding the window for completing questionnaires, the timeline for post-treatment follow-up, and changes to the dosage titration schedule.

### Eligibility criteria

Adults aged 18–65 may be eligible to participate in the LIFT if they meet the Canadian consensus criteria for ME/CFS and onset occurred prior to 2020. See Table 1 for full eligibility criteria. People who are treatment-naïve are prioritized. Participants are also asked to withhold the following medications for at least 1 month before beginning the study: “mitochondrial cocktail” (including coenzyme Q_10_, carnitine, creatine, riboflavin, alpha lipoic acid), midodrine, fludrocortisone, intravenous immunoglobulin, and Hizentra.

**Table 1:** Eligibility criteria

Inclusion criteria	Exclusion criteria
1. Meets National Academy of Medicine Criteria, Canadian Consensus Criteria, and demonstrates orthostatic intolerance for diagnosis of ME/CFS 2. 18–65 years of age at screening, inclusive 3. Onset of symptoms prior to December 2019 4. Female participant is not pregnant and at least 1 of the following conditions apply: a. Not a person of childbearing potential b. Person of childbearing potential who agrees to follow the contraceptive guidance from the time of informed consent 5. Agrees and can adhere to the study requirements for the length of the study 6. Body mass index (BMI) range of 17 to 40 kg/m^2^, inclusive 7. Agrees not to participate in another interventional study while participating in the present study 8. Has a functioning smartphone to download and run the DANA Brain Vital mobile application	1. Pregnant, planning to become pregnant, or breastfeeding 2. Any use of opioid medications within 30 days of screening 3. Positive urine test for opioids 4. History of alcohol, opioid, or other substance misuse 5. Participation in another interventional clinical trial in the last 30 days or planned during the trial period 6. Allergy to medication components 7. Any condition which, in the investigator’s opinion, makes the participant unsuitable for study participation 8. Diabetes mellitus (type 1 or 2) 9. Inpatient hospitalization within the 30 days prior to randomization or planned hospitalization or a surgical procedure during the study, which may affect the study assessments, in the opinion of the investigator 10. Clinically significant respiratory disease (such as chronic obstructive pulmonary disease, cystic fibrosis, severe asthma, lung infections including tuberculosis, sarcoidosis, thoracic endometriosis, pulmonary fibrosis, and lung cancers) and/or cardiac disease (medical history or current clinical findings) 11. Active malignancy or any other cancer 12. Initiated an exercise regimen within 4 weeks prior to randomization 13. Unable to read or write or physically unable to talk or write

### Sample size

With a sample size of 40 per trial arm (160 total participants), preliminary power calculations indicate the ability to detect moderate and large effects (≈>0.5 direct effect sizes) at 80% power and *α* = 0.05 from the two interventions under reasonable model assumptions [33–36]. The effect-size calculations refer to comparisons between each active-treatment arm and the double-placebo arm: low-dose naltrexone plus placebo versus double placebo, pyridostigmine plus placebo versus double placebo, and low-dose naltrexone plus pyridostigmine versus double placebo. However, the trial may not be powered to detect the synergistic interaction between the two medications, particularly after adjusting for multiple comparisons. To account for participant attrition, the trial will target enrollment of 180 patients.

### Recruitment, screening, and consent

Participants are recruited through two primary methods. Patients may be recruited through Dr. David Systrom’s or Dr. Donna Felsenstein’s clinics at Brigham and Women’s Hospital or Massachusetts General Hospital, respectively. Alternatively, potential participants may be recruited through the Open Medicine Foundation StudyME registry. Subjects recruited through StudyME are asked to provide access to their medical records for an initial prescreen. Medical records for all participants are used for inclusion/exclusion purposes, including screening for prior LDN and pyridostigmine use.

Potential participants are further screened during a visit to the Clinical Trials Hub at Brigham and Women’s Hospital. In addition to answering questions about the person’s medical history and signing the consent form, during the visit, research coordinators measure the subject’s height and weight, take vital signs, collect a urine sample to test for pregnancy and drugs of abuse, and set the subject up with a Garmin watch and Labfront and DANA Brain Vital accounts. Each participant is informed about their study status after lab results are returned and assessed by study staff, usually within 72 h.

Participants are provided with the informed consent form during the screening period, up to 28 days prior to their study visit initiating treatment. At the end of the screening period, coordinators contact the participant to confirm eligibility and answer questions about the consent form.

### Participant compensation

Participants are compensated for their time and effort via an Advarra payment card, which is based on activities completed:

- Screening visit (in person): $50
- Visit 1 (in person): $75
- Visit 2/3/4 (virtual): $25
- Visit 5 (in person): $75
- Visit 6 (virtual): $25

Subjects who complete the trial are also permitted to keep their Garmin watch.

### Interventions

LDN and pyridostigmine can be administered in many ways, with the most commonly used formulations being liquid and tablets [37, 38]. Both formulations are used clinically, though anecdotal reports from clinicians have indicated the liquid formulation is often better tolerated by patients. For this reason and to better allow up and down titration of drugs and placebo, this trial uses liquid formulations.

LDN and pyridostigmine are titrated up to their target doses over a period of 4 weeks. LDN is administered at 1.5 mg per day for the first 2 weeks and 3 mg per day for the following 2 weeks before reaching 4.5 mg per day for the remainder of the treatment period. Pyridostigmine follows a similar pattern, increasing from 20 mg three times per day to 40 mg three times per day to 60 mg three times per day (180 mg total daily) in 2-week intervals.

**Fig. 4:**
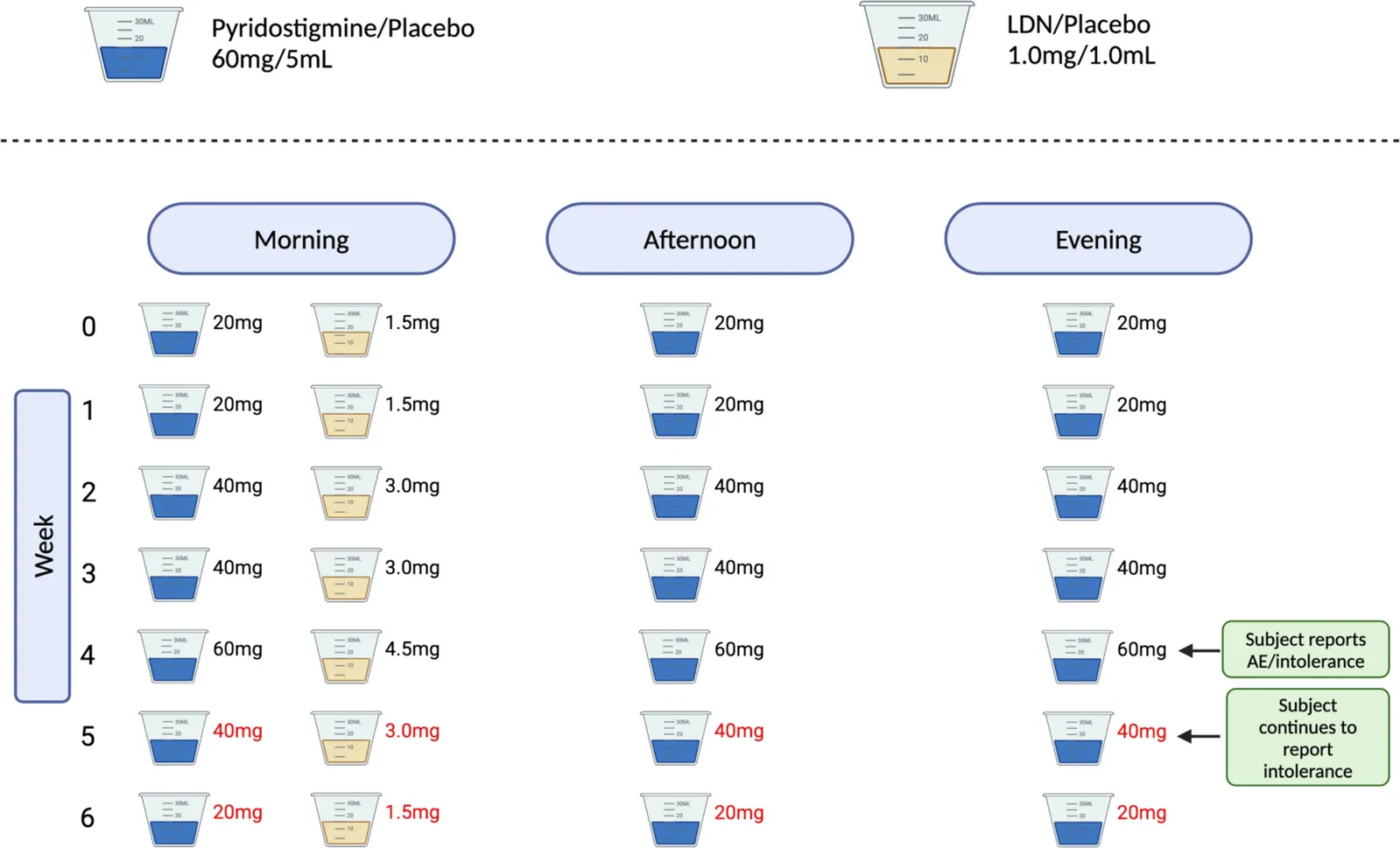
Titration schedule during treatment period, including intolerance plan. Participants will start at 20 mg pyridostigmine three times per day and 1.5 mg LDN in the morning for the first 2 weeks. For the next 2 weeks, those doses are doubled. Then, at week 4, the target dose is reached at 60 mg pyridostigmine three times per day and 4.5 mg LDN in the morning. If the participant reports an AE/intolerance, they will be moved back down to the previous dose (created in BioRender.com)

If a participant reports an adverse event or that they are not tolerating their current dose, the dose will be reduced to the previous level. See the full dosing schedule with intolerance plan in Fig. 4.

### Compliance monitoring

Participant compliance is monitored by coordinators via study visits, both in person and virtual. In addition, participants are asked to maintain a dosing diary throughout the treatment period, noting when they complete their daily doses. Missing a single dose is recorded as a minor deviation. Missing several doses sequentially is considered a major deviation and is documented via a breach report form that is promptly provided to the IRB.

### Adverse event monitoring

Coordinators perform an adverse event assessment during each in-person and virtual visit, taking place on weeks 2, 4, 10, 13, and 2 weeks following the treatment phase for follow-up. Participants are also given the principal investigator’s contact information so they can discuss anything they experience in between scheduled visits. If an adverse event is deemed severe and causally linked to the intervention, the participant may be unblinded before the completion of the trial.

### Outcomes

ME/CFS is a heterogeneous, multisystem disorder, and the two interventions are expected to affect partially distinct physiological pathways. Pyridostigmine may primarily influence autonomic and cardiorespiratory function, assessed by peak oxygen utilization, heart rate recovery, and oxygen uptake efficiency slope. Low-dose naltrexone may primarily influence neuroimmune and functional symptoms, assessed by FUNCAP-55. Multiple primary outcomes were therefore selected to capture both physiological and functional treatment effects. Multiplicity across these outcomes is addressed in the Statistical methods section.

The primary outcomes of the trial are FUNCAP-55 score, peak oxygen utilization, heart rate recovery, and oxygen uptake efficiency slope. Secondary outcome measures include cognitive testing, wearable device data, and symptom severity, as measured via surveys. FUNCAP-55 [39], PROMIS-29 [40], and DSQ-PEM [41] surveys are all validated measures. See Table 2 for the complete list of primary and secondary outcomes.

**Table 2:** Trial outcomes

Outcome measure	Source
Primary outcomes	
FUNCAP-55 score	Survey
Peak oxygen utilization	SHAPE test
Heart rate recovery	SHAPE test
Oxygen uptake efficiency slope	SHAPE test
Secondary outcomes	
PROMIS-29 score	Survey
DANA Brain Vital score	App
DSQ-PEM score	Survey
Step count	Garmin
Heart rate variability	Garmin
Heart rate	Garmin

Data will be stored on secure servers, and blood samples banked for future analyses will be identified by a coded ID number to preserve participant confidentiality.

### Auditing and data monitoring

The Trial Steering Committee is composed of three members of Open Medicine Foundation with strategic oversight of its research activities: the VP of Research Programs, the Chief Medical Officer, and the CEO. The primary coordinating center team is composed of the principal investigator (PI), three research coordinators, and a statistician. In addition, the coordinating center team includes three exercise physiologists who conduct the SHAPE tests and four co-investigators who assist with assessing eligibility, obtaining informed consent, performing blood draws, and managing medication orders.

The primary coordinating center team provides the Trial Steering Committee with biweekly reports of the progress of the trial, including notification of protocol amendments and occurrence of deviations. The trial PI also conducts a weekly review of the data, and the statistician will monitor data quality periodically throughout the trial. A Data Monitoring Committee was not engaged for this trial as the interventions are considered low-risk. Similarly, no interim analyses are planned.

### Statistical methods

Mixed effects models are an effective way to model data with multiple timepoints and subgroups, as is the case with the LIFT. The *mmrm* package in R [42] will be used for these data, which includes implementations of the Satterthwaite’s degrees of freedom method and Kenward-Roger approximation for *p *value estimation (which provide very similar results [43]). The model used will be a Mixed Model for Repeated Measures (MMRM), which is often used in clinical trials. The outcome measures are all continuous variables, and an unstructured covariance matrix will be used for the time dependence. Individual ID will be included as a random effect, as well as age, sex, and BMI as fixed effects. The main fixed effect of interest will be the interaction between group assignment and timepoint.

The testing for each hypothesis will determine whether the change in the given variable (see Table 2) across the timepoints is significantly different in the three case groups, relative to the controls. The significance level will be set at *p* < 0.05, adjusted for multiple tests using family-wise (Bonferroni) correction across the 12 primary tests (three groups, relative to the control group, with four outcomes for each). With 40 participants per group, a two-sided comparison at a Bonferroni-adjusted threshold of *α* = 0.05/12, an effect size of approximately *d* = 0.85 would be required to maintain 80% power. This strict threshold will reduce the probability of false positives, although potentially at the risk of some false negatives. The secondary outcomes will be tested in the same manner, stepwise. Hence, they shall be controlling for all primary and secondary tests, at the level of 12 + 6 × 3 = 30 tests. There will likely be some correlations, particularly between the questionnaire results, but this conservative level of correction will help to mitigate the risk of false positives.

Some missing data are anticipated, due to both dropouts and missed data entry. Mixed models are robust to missing data where the data are missing at random [44]. This may not be a reasonable assumption where there is a relationship between ME/CFS severity and missingness. This is discussed further in the Limitations section below.

Age and BMI, as two of the main covariates, shall be investigated and the distributions plotted as demographic variables, separated by sex. Associations for the covariates will be tested with linear models and are expected to be included in all primary analyses. If numbers are adequately balanced, the model will be run first with the whole model, and then with the data partitioned into male/female groups. This secondary analysis will only include age and BMI as the fixed effects.

A further secondary analysis will use machine learning classification techniques to identify the principal variables influencing responder/non-responder status to the intervention among the participants. Such variables will be drawn from the baseline information collected at the initial visit.

## Discussion

This report details the protocol for a 13-week randomized, double-blind, placebo-controlled clinical trial evaluating the efficacy of LDN and pyridostigmine in a pre-COVID-19 pandemic ME/CFS population. The study gathers physiological and symptom-based evidence of the impact of both drugs and will enable the investigation of potential predictors of response to each drug.

### Limitations

ME/CFS is an understudied disease, and this study is designed as an exploratory trial. Its primary purpose is to estimate treatment effects across several complementary clinical domains rather than to provide definitive evidence of efficacy for regulatory approval. Multiplicity is addressed as specified in the Statistical methods section. The findings will inform endpoint selection, effect-size estimates, sample-size calculations, and the overall design of future adequately powered confirmatory trials intended to provide definitive evidence of efficacy.

While the trial is preferentially enrolling treatment-naïve participants, the limited availability of such patients may require that some participants will be enrolled in the study having previous experience with one or both medications. In such an event, it is possible that the blinding for these participants may be compromised if they either respond or do not respond as they did in their previous experience. This risk is partially mitigated by the formulation of the drugs in the trial. The LDN, pyridostigmine, and placebo solutions are flavored to mask the taste of either of the drugs, which reduces the potential risk of a participant identifying their study arm assignment based on previous experience.

The lack of standardized outcome measures for ME/CFS research limits the ability to compare efficacy results from this trial with other studies. The primary outcomes for the LIFT were selected because they are objective measures of physiological response relevant to the potential mechanism of action of the drugs, as well as quasi-objective, validated survey measures of symptoms. Secondary outcomes include surveys that are frequently used in other research studies to facilitate comparisons where possible.

The use of the SHAPE test necessitates that participants are able to travel to the clinic, which excludes housebound and bedbound patients. Therefore, trial participants may differ from non-participants who otherwise fit the study inclusion criteria. Randomization mitigates the impact of a heterogeneous patient population, but the trial results may not be generalizable to ME/CFS patients of all severity levels.

Given the nature of ME/CFS, it is possible that disease severity and intolerance to the drugs being trialed will lead to data missingness. In addition, symptom fluctuation limits the ability to use common methods of statistically accounting for missing data (e.g., imputation). Therefore, the statistical power of the trial may be reduced. This risk is mitigated via regular check-ins with the study team to enhance compliance, as well as the incorporation of a 3-day window during which participants are able to complete surveys and cognitive assessments.

### Significance

The LIFT constitutes a significant contribution to the ME/CFS research field, with large potential implications for the future of clinical management of the disease. ME/CFS has no FDA-approved treatments. The LIFT is a randomized, double-blind, placebo-controlled trial, and as one of the largest trials ever conducted in the field, it is a major step forward toward providing patients with recognized, approved clinical treatment.

It is widely recognized that the ME/CFS patient population is heterogeneous, where each patient reacts differently to the same treatment strategy. This clinical trial acknowledges the heterogeneous nature of the patients and works to identify differences in responders and nonresponders. Identifying these differences has the potential to transform clinical practices, enabling a precision medicine approach to treatment strategies and ultimately improve outcomes.

## Trial status

Protocol version 04, June 24, 2024. Further modifications to the protocol that impact how the study is conducted will require an amendment, which will be approved by the Institutional Review Board and the trial funder will be notified prior to implementation. The protocol will also be updated in the clinical trial registry following the approval of each amendment. Recruitment began in August 2024 and is planned to be completed by May 2026.

## Supplementary Information

Supplementary Material 1. SPIRIT Checklist.

## Data Availability

Trial results will be published in an open access journal following completion of the study. The final trial dataset will be de-identified and made available in the public domain through an open access repository.

## Abbreviations

LDN: Low-dose naltrexone
LIFT: Life Improvement Trial
ME/CFS: Myalgic encephalomyelitis/chronic fatigue syndrome
SHAPE: Sub-maximum exercise heart and pulmonary evaluation
